# Abnormalities of Rest-Activity and Light Exposure Rhythms Associated with Cognitive Function in Patients with Mild Cognitive Impairment (MCI)

**DOI:** 10.5334/jcr.227

**Published:** 2023-12-28

**Authors:** Seong Jae Kim, Jung Hie Lee, Jae-Won Jang, Hee Seo Jung, In Bum Suh

**Affiliations:** 1 Department of Psychiatry, Chosun University College of Medicine, Gwangju, South Korea; 2 Department of Psychiatry, Chosun University Hospital, Gwangju, South Korea; 3 Department of Psychiatry, Kangwon National University School of Medicine, Chuncheon, South Korea; 4 Department of Psychiatry, Gwanggyo Good Sleep Clinic, Suwon, South Korea; 5 Department of Neurology, Kangwon National University School of Medicine, Chuncheon, South Korea; 6 Department of Psychiatry, Kangwon National University Hospital, Chuncheon, South Korea; 7 Department of Laboratory Medicine, Kangwon National University School of Medicine, Chuncheon, South Korea

**Keywords:** mild cognitive impairment (MCI), cognitive performance, rest-activity rhythm (RAR), light exposure rhythm (LER), nonparametric variables

## Abstract

We aimed to examine the difference in rest-activity rhythm (RAR) and light exposure rhythm (LER) between patients with mild cognitive impairment (MCI) and normal controls (NC), and to verify their relationships with cognitive functions. The neuropsychological battery was administered to participants above 50 years old. The MCI diagnosis was made according to Petersen’s criteria. Ten patients with MCI (77.90 ± 6.95 years) and eight NC (74.75 ± 5.06 years) were studied. Actigraphy (Actiwatch 2; Philips Respironics) was recorded at home for 5 days. RAR and LER variables, including interdaily stability (IS), intradaily variability (IV) and relative amplitude, were calculated using nonparametric analyses. The associations between cognitive performance and RAR and LER variables were explored using generalized linear models. There were no significant differences in RAR or LER variables between MCI and NC. There was a significant main effect of RAR-IS on the Stroop Color and Word Test (SCWT), indicating a positive relationship between RAR stability and SCWT performance. There was a significant group by RAR-IS interaction on Trail Making Test-A, indicating a negative relationship in MCI compared to NC. There was a significant group by LER-IV interaction on the Boston Naming Test, indicating a positive relationship in MCI compared to NC. There was no disruption in RAR and LER in patients with MCI. Our study showed that circadian rhythm abnormality was associated with a decline in executive function. However, circadian rhythm abnormality was not associated with declines in processing speed and language function in patients with MCI, implying an altered pathophysiology compared to NC.

## Introduction

Changes in circadian rhythms can be observed with aging, presumably due to aging of the circadian clock. These changes encompass alterations in physiological rhythms, such as melatonin and temperature [1]. Circadian rhythm disturbance is associated with various adverse health outcomes, including cardiovascular disease, diabetes, obesity, and cancer. These disruptions reportedly affect mortality in animal models [2]. Furthermore, circadian rhythm disturbance can lead to an acceleration of aging [3] and cause cognitive dysfunction in older individuals [4].

Nocturnal sleep disturbance in patients with Alzheimer’s disease (AD) may be primarily related to disruption of the rest-activity rhythm (RAR), which is a consequence of endogenous circadian rhythm disturbance. RARs tend to become more disrupted and irregular with age, which is more pronounced in AD [5]. It has been reported that greater RAR disturbance is associated with a larger cognitive decline in patients with AD, suggesting that this RAR disturbance may be another pathology associated with AD, further contributing to their cognitive decline [6,7]. RAR disturbance itself is associated with cognitive impairment [8].

Nonparametric methods for assessing the RAR may have good discriminative power in patients with AD [5]. Detrimental changes in the nonparametric RAR variables are often reported in patients with AD [9]. An increase in the intradaily variability (IV), indicating an increase in RAR fragmentation, is observed in patients with early-onset dementia [10] or even in those with preclinical AD [11]. Furthermore, a reduction in the relative amplitude (RA), indicating a decrease in RAR robustness, is consistently linked to the development of dementia or mild cognitive impairment (MCI) [7,12,13]. Reduced RAR robustness causes sleep and behavioral issues, such as a reversal of the day-night sleep pattern, particularly among patients with AD.

Light is an important entraining agent for endogenous circadian rhythms. Insufficient light exposure negatively impacts the RAR in older adults; the amount of daily light exposure is reportedly related to the circadian rhythm strength [14]. In turn, diurnal changes in the RAR might reflect those in the light exposure rhythm (LER). However, there is a limited number of studies that have examined the relationships between the RAR and LER using non-parametric methods, especially in patients with MCI [15]. Meanwhile, LER disturbance may be responsible for nocturnal sleep disturbance in patients in AD [19]. A previous study on nursing home patients who were exposed to light levels above 1,000 lux for only 10.5 minutes per day, found a relationship between nighttime awakenings and low illumination levels [16].

The conversion rate from MCI to AD is approximately 10–15% per year. The cognitive domains of memory and attention/executive deficits are at risk in AD [17]. A recent meta-analysis shows that the cognitive domains of verbal memory and language ability are highly accurate in predicting the conversion from MCI to AD [18]. Whitwell et al. [19] found that 70% of patients with MCI with executive deficits progressed to dementia within 4 years. These patients showed atrophy of the basal forebrain and hypothalamus, a region that is a crucial regulator of the circadian rhythm. Therefore, it has been hypothesized that circadian rhythm disturbance occurs concurrently with a decline in executive function, contributing to the development and progression of dementia [20,21]. Okuda et al. [22] determined that RAR irregularity is associated with a decline in executive function and working memory in older people. However, the vulnerability of a specific cognitive domain to RAR disturbance in patients with MCI remains undetermined.

We aimed to compare the characteristics of RAR and LER, measured using a nonparametric approach, between patients with MCI and normal controls (NC) in a community setting. Additionally, we aimed to examine the relationship between the nonparametric variables of the rhythms and multiple cognitive domains in patients with MCI. We hypothesized that the RAR and LER would differ between patients with MCI and NCs, which would be reflected as changes in a specific cognitive domain.

## Methods

The study protocol was approved by the Ethical Board of Kangwon National University Hospital (No: KNUH-2019-04-003; March 2, 2020) and was conducted in accordance with the principles of the Declaration of Helsinki. Written informed consent was obtained from each participant and their legal representative prior to being enrolled in the study.

### Participants

Patients with MCI were recruited from the Dementia Clinic at Kangwon National University Hospital and two Public Centers for Dementia Care. MCI was diagnosed by a psychiatrist or a neurologist according to Petersen’s criteria [23]. Petersen’s criteria include the presence of subjective or objective memory impairment without dementia and a score more than 1.5 standard deviations lower than the normative value in at least one of the neurocognitive tests using the Korean version of the Consortium to Establish a Registry for Alzheimer’s Disease (CERAD-K) packet [24]. Normal controls (NCs) were recruited through local health care centers. NCs were defined as those with no evidence of cognitive impairment, whose neurocognitive test scores for each cognitive domain were 1.0 standard deviation below the mean score or higher. The exclusion criteria were as follows: 1) current substance-related disorders, depressive disorders, or other psychiatric disorders diagnosed using the DSM-5; 2) conditions affecting their circadian rhythms (e.g. shift work, jet lag); 3) current use of any medications affecting sleep and circadian rhythms; 4) diagnosis of a primary sleep disorder; and 5) current medical illness, including liver cirrhosis, chronic pulmonary disease, cancer, uncontrolled diabetes, or uncontrolled hypertension.

### Procedures

Four of the referred participants were excluded because they were taking medication that affects sleep and circadian rhythms. Ten of them declined to participate in the study. The enrolled patients were further evaluated with self-report questionnaires, including the Korean versions of the Morningness-Eveningness Questionnaire (MEQ-K) [25], Epworth Sleepiness Scale [26], Beck Depression Inventory (BDI-K), and the Pittsburgh Sleep Quality Index (PSQI) [27]. Ten patients with MCI (77.90 ± 6.95 years; male: female = 5:5) and eight NCs (74.75 ± 5.06 years; male: female = 2:6) were finally selected. No participants had a BDI-K score of 19 or higher, which is clinically suggestive of depression. Participants with a KESS score >11 were further evaluated for primary sleep disorders by a sleep physician through a phone screening interview, and there were no clinical findings to suggest they had sleep disorders.

Subsequently, actigraphy monitoring using an actiwatch with an integrated light sensor (Actiwatch 2; Philips Respironics, Murrysville PA, USA) was conducted at home for five consecutive days. Participants also completed a sleep diary. Participants were instructed to wear the actigraph continuously and to avoid covering the light sensor with clothing. They were required to abstain from alcohol consumption during actigraphy monitoring. To avoid including artifact data due to a sleeve covering the light sensor, light data below 1 lux during an active interval of actigraphy were removed from the analysis. Light exposure data were log-transformed based on their highly skewed empirical distribution, while raw data for the rest-activity were used [28].

### Neuropsychological Assessment

Neurocognitive function was assessed using the CERAD-K Neuropsychological battery, which consists of the following 10 tests: verbal fluency (VF), a modified Korean version of the Boston Naming Test (BNT), the Korean version of the MMSE (MMSE-KC), word list memory (WLM), constructional praxis (CP), word list recall (WLR1), word list recognition (WLR2), constructional recall (CR), trail making test-A (TMT-A), and Stroop Color and Word test (SCWT). The raw neurocognitive test scores and TMT-A completion time were transformed to z-scores adjusting for age, sex, and years of education. The z-scores were subsequently used in our statistical analyses.

### Actigraphy

Actigraphy data were derived using the Actiware-Sleep Software (version 6.0.2; Philips Respironics, Murrysville, PA, USA). The instructions for wearing the Actiwatch and guidelines for the qualitative assessment of the collected data have been previously described [28]. The following sleep parameters were calculated based on the sleep period from light off to light on described in their sleep diaries: time in bed, total sleep time, sleep onset, sleep-onset latency, wake time after sleep onset, sleep efficiency, and fragmentation index. The sleep parameters for one patient with MCI could not be obtained due to an unexpected software error.

Rest-activity and light exposure data were computed into hourly bins. Data with hourly bins across 5 days were then used for nonparametric analysis. If data within a bin was missing, the value for the bin was interpolated using the mean of the values before and after the missing bin [28]. Nonparametric analyses of each RAR and LER were performed using R statistical software (package “narACT”) [29]. Rest-activity and light exposure data were computed into hourly bins. The variables for RAR and LER, including interdaily stability (IS), IV, and RA, were calculated using nonparametric analyses. The IS quantifies the stability between days. IV quantifies the frequency and extent of transitions between rest and activity. Data from the most active 10-h period (M10) and the least active 5-h period (L5) were used to calculate RA using the following formula: RA = (MI0 – L5)/(MI0 + L5).

### Statistical analysis

Demographic data and the scores of K-BDI-II, PSQI, KESS, and MEQ-K were compared between the MCI and NC groups using the Chi-squared test or Mann–Whitney U test. The neurocognitive test scores, sleep parameters, and nonparametric RAR and LER variables of the MCI and NC groups were compared using the Mann–Whitney U test.

Generalized linear models were conducted to evaluate the main and interaction effects of group and each nonparametric variable on each cognitive function. The group (MCI vs. NC) and nonparametric variables (IS, IV, and RA) were considered independent variables and each cognitive function, which had significant group differences, was considered the dependent variable. The Shapiro–Wilk test was applied to each dependent variable to determine the model type based on distribution. All statistical analyses were performed using SPSS (version 18.0; SPSS Inc, Chicago, IL, USA).

## Results

### Demographic and clinical characteristics

There were no significant differences in age, sex ratio, or education level between the MCI and NC groups. There were no significant differences in the K-BDI, PSQI, KESS, or MEQ-K scores between the two groups (Table 1).

**Table 1:** Demographic and clinical characteristics in the MCI (N = 10) and NC (N = 8) groups.


	MCI GROUP	NC GROUP	P

Amnestic: Non-amnestic	7:3		

Age (years)	77.90 ± 6.95	74.75 ± 5.06	.349

Gender (M: F)	5:5	2:6	.280

Education(years)	7.90 ± 5.95	8.50 ± 5.07	1.00

K-BDI-II	5.40 ± 5.30	8.50 ± 2.98	.179

PSQI	5.70 ± 2.91	6.75 ± 2.38	.530

KESS	6.00 ± 4.16	6.50 ± 4.75	.654

MEQ-K	65.20 ± 8.07	65.00 ± 5.63	.929


Mann-Whitney U test or X^2^ test. Data are shown as mean ± SD or ratio. MCI: mild cognitive impairment, NC: normal controls, K-BDI-II: Korean version of the Beck Depression Inventory-II, PSQI: Pittsburgh Sleep Quality Index, KESS: Korean version of the Epworth Sleepiness Scale, MEQ-K: Korean version of the Morningness-Eveningness Questionnaire.

The scores of VF, BT, WLM, and SCWT were significantly lower and TMT-A completion time was significantly longer in the MCI group than in the NC group (*p* < 0.05) (Table 2).

**Table 2:** Neurocognitive functions† in the MCI (N = 10) and NC (N = 8) groups.


	MCI GROUP	NC GROUP	P^†^

VF	9.70 ± 2.45**	17.50 ± 5.13	.002

BNT	9.50 ± 2.12*	12.50 ± 1.77	.011

MMSE-KC	21.70 ± 4.99	25.75 ± 3.33	.061

WLM	10.70 ± 5.798*	18.12 ± 3.52	.010

CP	9.40 ± 1.84	9.88 ± 1.64	.674

WLR1	3.60 ± 2.27	5.38 ± 2.20	.128

WLR2	8.20 ± 2.20	9.12 ± 1.46	.317

CR	5.90 ± 2.28	6.12 ± 3.83	.893

TMT-A time (sec)	149.29 ± 108.49 (n = 7)*	69.38 ± 15.49	.032

SCWT	15.29 ± 8.42 (n = 7)*	28.38 ± 10.25	.037


* p < 0.05, ** p < 0.01 (^†^: Mann-Whitney U test for z-scores adjusted for age, sex, and education). Data are shown as mean ± SD. MCI: mild cognitive impairment, NC: normal controls, VF: verbal fluency, BNT: Boston naming test, WLM: word list memory, WLR1: word list recall, CP: constructional praxis, WLR2: word list recognition, CR: constructional recall, TMT-A: trail making test-A, SCWT: Stroop color-word test.

### Sleep parameters, RAR, and LER

There were no significant differences in the actigraphy-measured sleep parameters between the MCI and NC groups (Table 3). There were no significant differences in the RAR or LER variables (IS, IV, or RA) between the MCI and NC groups (Table 4).

**Table 3:** Sleep parameters in the MCI (N = 9) and NC (N = 8) groups.


	MCI GROUP	NC GROUP	P

Time in bed (h)	8.62 ± 1.53	8.16 ± 0.93	.386

Bedtime (h:m)	21.60 ± 0.79	21.92 ± 0.41	.630

Wake time (h:m)	6.23 ± 1.23	6.14 ± 1.01	.847

Total sleep time (h)	6.22 ± 1.78	6.16 ± 0.94	.773

Sleep-onset latency (min)	22.37 ± 22.87	19.85 ± 22.38	.700

Sleep onset (h:m)	21.97 ± 0.70	22.23 ± 0.56	.700

Sleep efficiency (%)	71.47 ± 12.62	75.15 ± 9.33	.531

Wake time after sleep onset (min)	91.06 ± 42.52	81.67 ± 31.07	.501

Fragmentation index	47.85 ± 19.50	43.68 ± 11.43	.773


Mann-Whitney U test. Data are shown as mean ± SD. MCI: mild cognitive impairment, NC: normal controls.

**Table 4:** Nonparametric variables of the rest-activity and light exposure rhythms in the MCI and NC groups.


	MCI GROUP (N = 10)	NC GROUP (N = 8)	P

***Rest-activity rhythm***			

IS	0.61 ± 0.07	0.60 ± 0.10	.859

IV	0.77 ± 0.32	0.88 ± 0.12	.168

RA	0.86 ± 0.10	0.87 ± 0.81	.859

***Light exposure rhythm***			

IS	0.67 ± 0.14	0.68 ± 0.09	.964

IV	0.51 ± 0.25	0.61 ± 0.20	.196

RA	0.93 ± 0.16	0.95 ± 0.09	.854


Mann-Whitney U test. Data are shown as mean ± SD. MCI: mild cognitive impairment, NC: normal controls, IS: interdaily stability, IV: intradaily variability, RA: relative amplitude.

### Effects of RAR and LER on neurocognitive functions

**Figure 1:**
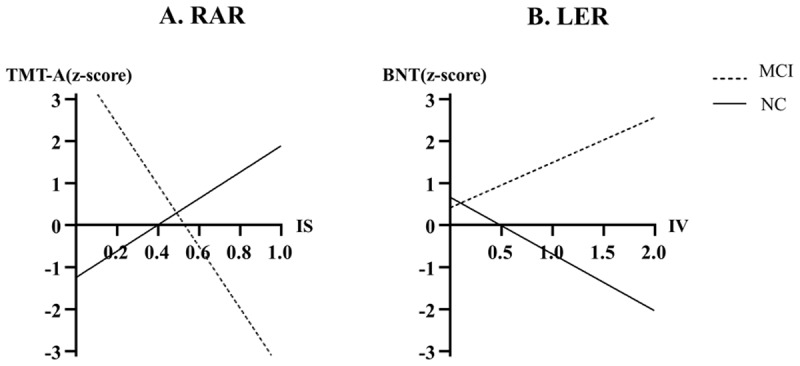
Group by RAR-IS significant interaction predicting z-scores of TMT-A **(A)**, and group by LER-IV significant interaction predicting z-scores of BNT **(B)** in the generalized linear models. The dotted lines indicate z-scores of TMT-A by changes of the RAR-IS **(A)**, and z-scores of BNT by changes of the LER-IV **(B)** in the MCI group, respectively. Likewise, the solid lines indicate those respective scores in the NC group. NC: normal control, MCI: mild cognitive impairment, TMT-A: Trail Making Test-A type, BNT: Boston Naming Test, RAR: rest-activity rhythm, LER: light exposure rhythm, IS: interdaily stability, IV: intradaily variability.

A significant group by RAR-IS interaction was observed on the TMT-A (β = –10.45, *p* < 0.05) (Table 5); there was a negative relationship between RAR-IS and TMT-A z-scores in the MCI group and a positive relationship in the NC group (Figure 1A). There was a significant main effect of IS on the SCWT z-scores (β = 6.32, p < 0.05); higher IS was associated with a higher SCWT score.

**Table 5:** Main effects of nonparametric variables of rest-activity rhythm and group by interactions on VF, BNT, WLM, TMT-A and SCWT (N = 18).


	*IS*	*GROUP BY IS*	*IV*	GROUP BY IV	*RA*	*GROUP BY RA*
		
**VF**						

**β (95% CI)**	3.30 (–2.5~9.1)	0.54 (–9.0~10.1)	1.23 (–4.0~–6.4)	–0.32 (–5.8~5.1)	–5.9 (–13.4~1.5)	6.65 (–2.6~15.9)

**BNT**						

**β (95% CI)**	1.63 (–2.4~5.6)	1.90 (–4.6~8.41)	0.93 (–2.7~4.57)	–1.23 (–5.1~2.6)	–3.52 (–8.7~1.7)	3.13 (–3.3~9.6)

**WLM**						

**β (95% CI)**	–2.76 (–10.7~5.1)	5.30 (–7.6~18.2)	3.34 (–2.9~9.6)	–1.36 (–7.9~5.2)	–1.65 (–11.8~8.5)	4.35 (–8.3~17.0)

**TMT-A†**						

**β (95% CI)**	3.14 (–2.7~9.0)	–10.45* (–19.9~**–1.0)**	–3.12 (–8.2~2.0)	4.58 (–0.8~9.9)	0.42 (–7.5~8.3)	–5.51 (–15.6~4.6)

**SCWT**						

**β (95% CI)**	6.32* **(1.1~11.5)**	–7.56 (–16.3~1.2)	–3.52 (–8.5~1.5)	3.01 (–3.7~9.7)	0.79 (–7.06~8.63)	–1.33 (–12.65~9.99)


* p < .05 (Generalized linear model), ^†^:(n = 17). IS: interdaily stability, IV: intradaily variability, RA: relative amplitude, VF: verbal fluency, BNT: Boston naming test, WLM: word list memory, TMT-A: trail making test-A, SCWT: Stroop color-word test.

A significant group by LER-IV interaction was observed on the BNT z-scores (β = –2.43, p < 0.05) (Table 6); there was a positive relationship between LER-IV and BNT z-scores in the MCI group and a negative relationship in the NC group (Figure 1B). The IS and RA did not have any significant main effects, and there were no significant interactions between group and those variables on the z-scores of the VF, BNT, WLM SCWT, or TMT-A.

**Table 6:** Main effects of nonparametric variables of light exposure rhythm and group by them interactions on the VF, BNT, WLM, TMT-A and SCWT (N = 18).


	*IS*	*GROUP BY IS*	*IV*	*GROUP BY IV*	*RA*	*GROUP BY RA*
		
**VF**						

**β (95% CI)**	–1.12 (–8.4 ~ 6.2)	1.26 (–7.1~9.7)	2.56 (–0.29~5.4)	–1.63 (–5.1~1.9)	–4.70 (–11.4~2.0)	4.17 (–3.3~11.7)

**BNT**						

**β (95% CI)**	–0.62 (–5.4~4.2)	2.50 (–3.0~8.0)	1.08 (–0.8~3.0)	–2.43* (–4.8~**–0.1)**	–1.57 (–6.3~3.2)	1.95 (–3.4~7.3)

**WLM**						

**β (95% CI)**	–6.51 (–15.2~2.2)	3.31 (–6.7~13.3)	2.16 (–1.3~5.6)	0.81 (–3.5~5.1)	0.58 (–8.5~9.6)	1.16 (–89.0~11.3)

**TMTA†**						

**β (95% CI)**	–0.95 (–8.70~6.8)	1.87 (–7.2~11.0)	1.23 (–2.0~4.4)	–2.32 (–6.3~1.7)	–0.89 (–7.4~5.6)	4.87 (–2.4~12.2)

**SCWT**						

**β (95% CI)**	1.31 (–5.9~8.6)	–1.29 (–9.8~7.3)	0.95 (–2.1~4.0)	–1.05 (–5.0~2.9)	2.59 (–4.2~9.4)	–1.67 (–9.3~6.0)


* p < .05 (Generalized linear model), ^†^:(n = 17). IS: interdaily stability, IV: intradaily variability, RA: relative amplitude, VF: verbal fluency, BNT: Boston naming test, WLM: word list memory, TMT-A: trail making test-A, SCWT: troop color-word test.

## Discussion

We found no differences in the RAR and LER characteristics between patients with MCI and NCs (Table 4). Our results are consistent with those of a previous study [15], where no significant differences in the IS, IV, and RA of RAR or LER were found between 21 patients with MCI and age-matched controls. These findings suggest that the RAR or LER may not be sensitive enough to differentiate patients with MCI from normal older adults. These findings contradict those of another study [30], in which the RA of the RAR was lower in the MCI group compared to the control group.

As sleep disturbance may be a risk factor for cognitive decline, it has been hypothesized that patients with MCI would exhibit more sleep disturbance than normal controls [31]. However, we found no differences in objective sleep quality between the two groups, based on sleep parameters from actigraphy (Table 3). Kim et al. [28] reported an association between the RAR with sleep efficiency and wake after sleep onset in patients with insomnia. From this perspective, our finding of no difference in objective sleep quality between the two groups may reflect a lack of group differences in the RAR.

Clinical characteristics that may affect cognitive function, such as depressive symptoms, subjective sleep quality, and daytime sleepiness, did not differ significantly between patients with MCI and NCs (Table 1). Patients with MCI exhibited deficits in multiple cognitive domains, including executive function, language function and verbal memory, compared to NCs (Table 2). It is known that executive function contributes to the development and progression of dementia [20,21]. In addition, language impairment has been implicated as an early sign of progression to AD [32].

We further evaluated whether cognitive deficits in patients with MCI could be explained by the degree of RAR or LER disruption. Generalized linear models for the RAR parameters revealed that the IS was positively associated with SCWT performance, with no group interaction on the test. This suggests that the IS of the RAR decreases with declining executive function in the elderly, regardless of the presence or absence of cognitive impairment. If the IS of the RAR is reduced, it would be manifested in an irregularity of sleep-wake rhythm in the real world. Our finding is similar to the findings of the study by Alfini et al. [30], which reported that RAR variables with positive connotations, such as higher IS, lower IV, and higher RA, were associated with better executive function across groups, including patients with MCI and controls. Okuda et al. [22] also reported that the regularity of sleep timing was associated with better performance in executive function and working memory in the elderly.

In generalized linear models, we found that higher IS was associated with longer TMT-A completion times in patients with MCI, reflecting lower processing speed performance. In contrast, a higher IS was associated with shorter TMT-A completion times in NCs, reflecting higher processing speed performance (Fig.1A). A higher IS indicates the extent of consistency of RARs across days, and is considered a measure with a positive connotation. Given this, the finding in patients with MCI are unexpected. The finding is obviously limited in rationale, however, suggesting that, unlike NCs, the stability of RARs over days in patients with MCI is not better explained by an increase in simple processing speed. A large cohort study of healthy older adults found that a longitudinal change in global cognition positively correlated with longitudinal changes in IS [21], which is parallel with our findings indicating that increases in IS are associated with improved executive functions, including the SCWT and TMT-A in NCs. To the best of our knowledge, no study has previously verified the relationship between RAR and executive functions in patients with MCI. Given that deficits in executive functions may contribute to the development of dementia [21], our findings in the elderly, including in patients with MCI, showing a relationship between IS and decline in executive functions are meaningful.

Generalized linear models of LER revealed a positive relationship between IV and BNT performance in patients with MCI, in contrast to the relationship observed in NCs (Fig.1B). A higher IV of LER, which reflects increased exposure to light during sleep, may be associated with sleep disruptions [33]. In this regard, our finding that an increase in the IV is linked to an improvement in language function in patients with MCI seems counterintuitive. As a plausible explanation for this finding, LER disturbance in patients with MCI might not reflect the expected degree of language impairment. Nonetheless, this relationship in patients with MCI requires further study.

To the best of our knowledge, no previous studies have verified the relationship between LER and cognitive function. A recent study on college students suggests the possibility that light patterns are mirrored by irregular sleep schedules, which are associated with lower academic performance [34]. Given that language dysfunction has been implicated as an early biomarker of AD [32], our study findings may be clinically significant.

Our study had some limitations. First, the study population was relatively small, which does not provide adequate power to evaluate our outcomes. Second, our controls were not selected as an age- and gender-matched sample of patients with MCI, thus differences in age and sex between the two groups may act as a potential confounding factor. Third, our battery of neuropsychological tests may not fully cover the range of cognitive domains affected in patients with MCI. In future studies, a broader battery of neuropsychological tests would be required to provide a more comprehensive evaluation of cognitive functions, and to verify their relationship with RAR and LER.

Despite these limitations, to the best of our knowledge, this is the first study that has examined the relationships between changes in the RAR and LER and the cognitive function in patients with MCI. Furthermore, some potential interventions, such as light therapy or targeted cognitive training, can help to improve circadian rhythms or mitigate cognitive decline in patients with MCI.

In conclusion, the day-to-day stability of RAR in our MCI group exhibited a positive correlation with the SCWT performance. However, the day-to-day stability of RAR did not positively correlate with the TMT-A performance, which differed from findings of NCs. Furthermore, the fragmentation of LER did not negatively correlate with the BNT performance. These findings suggest that rest-activity or light exposure rhythm abnormalities in patients with MCI may reflect a decline in executive function, but not necessarily a decline in cognitive processing speed or language functions.
